# Changes in spending, quality indicators, and provider experiences following the introduction of a population-based payment model in dutch primary care: a mixed methods evaluation

**DOI:** 10.1007/s10198-025-01765-8

**Published:** 2025-03-11

**Authors:** Tadjo Gigengack, Daniëlle Cattel, Frank Eijkenaar

**Affiliations:** 1 Erasmus School of Health Policy and Management, Erasmus University Rotterdam, Rotterdam, The Netherlands; 2 Erasmus Centre for Health Economics Rotterdam, Erasmus University Rotterdam, Rotterdam, The Netherlands

**Keywords:** Payment reform, Impact evaluation, Population payment, Healthcare expenditure, Reimbursement mechanism, The Netherlands, I11, I13, I18, H51, D04

## Abstract

**Background:**

In July 2017, a Dutch health insurer and primary care organization jointly implemented the All-In Contract (AIC), a population-based payment model for general practitioners (GPs). Affiliated GP-practices received a capitated payment per enrolled patient covering all GP care and multidisciplinary primary care for chronic conditions. Additionally, the care organization shared in savings and losses on total healthcare spending, contingent upon meeting quality targets. This study investigates the AIC’s impact on spending, quality indicators, and provider experiences 2.5 years after implementation.

**Methods:**

We employed a difference-in-differences approach comparing individual-level claims spending from enrollees of participating GP-practices (N = 16,425) with a control group (N = 212,251). Changes in indicators of chronic care management and patient satisfaction were investigated in a before-after analysis due to limited data availability. To contextualize the findings and explore provider experiences, focus groups were conducted with stakeholders involved in the development and/or implementation of the AIC.

**Results:**

The AIC was associated with an insignificant 1.2% reduction of average quarterly total spending per enrollee (p = 0.476). We did find a − 10.2% decrease in primary care spending growth (p < 0.01), which was likely related to the indexation rate used for the capitation payment. Spending in other subcategories showed insignificant changes. Changes in patient satisfaction and chronic care management indicators were mixed and modest, but due to the lack of data from non-participating GPs, the extent to which these changes can be attributed to the AIC remains uncertain. The focus group participants reported improvements in provider flexibility in care provision, autonomy, and reduced administrative burdens. However, the focus group results may not fully capture the broader or more diverse experiences of all providers involved.

**Conclusions:**

In its first 2.5 years, the AIC had no significant effect on total healthcare spending growth. Trends in quality indicators suggest mixed results for patient satisfaction and chronic care management, while focus group results indicated improved provider experiences. To comprehensively evaluate population-based payment reforms, stakeholders should improve data collection strategies to enable causal assessment of population health, patient experiences, and provider well-being.

## Introduction

Spending on healthcare services in OECD countries has grown rapidly over the past 50 years, increasing from an average of 4.6% of gross domestic product (GDP) in 1970 to 8.8% in 2019 [1]. Consequently, healthcare purchasers, policymakers, and other stakeholders have increasingly been grappling with the challenge of containing healthcare spending growth without compromising access to and quality of care. One promising strategy that can contribute to addressing this challenge is provider payment reform.

The rationale for provider payment reform is twofold. First, it is well-established that financial incentives and changes in those incentives can have a significant impact on provider behavior in terms of the volume and quality of care provided [2, 3]. For instance, studies conducted in the United States (US) and the Netherlands have shown that general practitioners (GPs) paid by fee-for-service (FFS) generally provided more care than GPs who were (partly) salaried or capitated [4–6]. Second, the incentives in predominant provider payment models are often poorly aligned with the overarching goals of healthcare systems, as often described by the quadruple aim [7].

The quadruple aim is a framework for delivering high-value care that emphasizes simultaneously improving (i) experiences of patients, (ii) health of populations, (iii) affordability, and (iv) experiences of healthcare professionals [7]. Predominant payment models tend to emphasize volume over value, which can lead to overprovision of care and limited efforts directed at quality improvement, prevention, and care coordination [8, 9].

Provider payment reform aims to align financial incentives with the principles of the quadruple aim. A promising example is population-based payment [10–15]. Under this model, care organizations receive a prospective payment per registered individual and/or face spending targets within an existing FFS structure [16]. Providers who manage spending below the target may retain or share the savings, while exceeding the target may result in shared losses. Performance on quality measures often determines the extent of financial rewards or penalties [8, 16]. In addition, depending on the exact design, this payment approach may also improve patient and provider experiences by offering more room and flexibility in care provision, allowing more time for patient care, and reducing administrative burdens. Population-based payment initiatives often center on GPs, focusing on shifting care from specialist to primary care settings [13].

Population-based payment initiatives often assign a central role to GPs and emphasize shifting care from medical specialist care to primary care [13]. Most of these initiatives originate from the US [10, 11, 13], but they have also become more prevalent in Europe [14, 17]. Evidence suggests such models have potential to slow spending growth while maintaining care quality [13, 18]. However, much of this evidence comes from the US, leaving limited insight into their effectiveness in other contexts. More research is needed on the impacts of population-based payment on achieving the quadruple aim, particularly outside the US [13].

This study evaluates the impact of a primary care population-based payment initiative in the Netherlands—the All-In Contract (AIC). Implemented in July 2017, the AIC was developed by a large Dutch health insurer and a nationally operating primary care organization encompassing multiple GP practices. The AIC constituted a meaningful change in the structure and complexity of the payment system of Dutch GPs. In the Dutch healthcare system, GPs are the first point of contact for patients, coordinate care for patients with chronic conditions, and act as gatekeepers to medical specialist care. For GPs to effectively fulfill these roles, it is essential to have financial incentives in place that are aligned with these roles [17]. However, the regular payment model for Dutch GPs, the so-called the three-segment model, is widely considered to fall short in this regard.

The three-segment model is a blend of different payment models, including a fixed capitation fee and FFS payments for consultations and certain procedures and tests (segment 1), bundled payments for some chronic conditions (segment 2), and pay-for-performance provisions for quality, efficiency, and innovation (segment 3). Despite its intention to promote high-value care, this model is often criticized to incentivize volume over prevention or cost-effective referrals [17]. Furthermore, its complexity and extensive list of claim codes also create significant administrative burdens and limits the use of innovative ways of care delivery without predefined claim codes [19].

The AIC replaces the three-segment model with a simplified payment model comprising two components. The first component – the capitation payment or ‘all-in tariff’ – provides a quarterly payment covering all GP care plus multidisciplinary primary care for selected chronic conditions. This design aims to eliminate volume incentives, reduce administrative burdens, and support innovative care models such as e-health. However, capitation may also incentivize underutilization and raise concerns about incentives for selective patient enrollment and referrals [20–22]. To address this, a second component—the shared savings/losses model—was introduced. This model holds the participating organization financially accountable for health care quality and total healthcare spending, including hospital care, thereby encouraging efficient referrals and substitution of hospital care with primary care [17].

By assessing how GPs participating in the AIC responded to the new incentives during its first 2.5 years, this study contributes to the literature in two ways. First, by evaluating a population-based payment initiative in the context of Dutch primary care, we add to the evidence on the effects of population-based payment beyond the context of the US healthcare system. Second, by using various data sources and combining quantitative analyses of changes in spending and quality with a qualitative analysis of stakeholder experiences, we obtain a comprehensive understanding of the AIC’s impact.

This paper is structured as follows: Sect. "Study setting and intervention" describes the study setting and intervention in detail. Sect. "Data and Methods" outlines data sources and methodology. Sect. "Results" presents the results, followed by a discussion and summary in Sect. "Discussion".

## Study setting and intervention

### The Dutch healthcare system and the role of GPs

The Dutch healthcare system for curative care operates on the principles of managed competition [20]. Healthcare providers compete for favorable contracts with competing private health insurers acting as prudent buyers of care on behalf of their enrollees within regulatory limits set by the government. These limits include mandatory coverage for a standardized basic benefits package, annual open enrolment, community-rated premiums, and risk equalization [23]. In addition, consumers can voluntarily choose to purchase supplementary health insurance coverage, for which insurers are free to set the terms of the insurance contract [24]. The Dutch health insurance market consists of four major health insurers with nationwide provider networks and several smaller insurers with more limited market shares. The four largest insurers together represent around 84% of the total market [25].

GPs play a critical role in the Dutch healthcare system. They serve as the initial point of contact for patients with new health problems and coordinate the provision of chronic care services. In addition, GPs act as gatekeepers to non-emergency specialist care services, requiring patients to obtain a referral before accessing these services. Emergency specialist care, such as emergency room visits, does not require a referral. The Dutch system for primary care is characterized by a high rate of patient registration with a GP for long periods of time, with 90% of patients enrolled for over 2 years with the same GP and two-thirds for over 10 years [19]. Over the past years, more GPs have been working in group practices or larger health centers, alongside other primary care professionals such as nurses, physical therapists, and psychologists [26]. These practices and centers can be either independently owned or part of a larger primary care organization. Due to the so-called ‘following policy’ (in Dutch: volgbeleid), agreements made by the market-leading insurer in a region typically apply to all patients of a GP practice. Other insurers adopt the similar contracts for that practice, ensuring consistency across the patient population [28].

### Regular payment model for Dutch GPs: the three-segment model

Currently, GPs in the Netherlands are reimbursed through a blended payment model comprising three segments [19]. Segment 1 (S1) pertains to basic GP services, consisting of a combination of a quarterly capitation fee for each enrolled patient and more than 70 unique billable codes for consultations, diagnostic tests, and medical treatments [19]. The capitation fee is adjusted for patient age (four classes) and whether patients reside in a deprived area (yes/no). In addition, S1 covers reimbursement of mental health assistants in primary care (in Dutch: POH-GGZ) [19]. S1 accounts for approximately 70% of total GP reimbursement [27].

Segment 2 (S2) covers multidisciplinary primary care that, in addition to GPs, also involves other disciplines to provide integrated care for patients with specific chronic conditions [19]. This segment consists of three parts: S2A, S2B, and an additional module for financing infrastructural and organizational changes that are required for multidisciplinary collaboration in primary care [19, 29]. S2A consists of bundled payments for four chronic conditions: diabetes mellitus type 2 (DM2), Chronic Obstructive Pulmonary Disease (COPD), asthma, and vascular risk management (VRM). S2B covers care activities that are not part of the other elements of S2. This can concern either new bundles for other conditions, or innovations in care activities within existing bundles (e.g., newly developed diagnostic treatments). S2 accounts for approximately 15% of total GP reimbursement [24].

Finally, segment 3 (S3) offers room for explicitly rewarding performance and innovation [19]. Examples of areas for which performance indicators can be defined are efficient diagnostics and drug prescribing; service and accessibility; and quality of chronic care delivery that is part of S2 [19]. Examples of primary care innovation include e-health and e-consultation of medical specialists (i.e., calling on expertise from specialized providers without having to make a referral). Depending on the contractual agreements made with insurers, S3 accounts for approximately 15% of total GP reimbursement [27].

Despite the original intention to facilitate GPs in delivering high-value care, the three-segment model inadvertently encourages the volume of procedures and consultations, while failing to incentivize prevention or cost-effective referrals [17]. Furthermore, the multitude of payment models combined with a vast array of claim codes is perceived as complex, can impose significant administrative burdens on GPs, and restrict the use of innovative care services without billable claim codes [19].

### Intervention under study: All-In Contract (AIC)

Following the introduction of the pilot, in July 2017 the insurer and primary care organization implemented the AIC. With this payment contract, the three-segment model was replaced by a population-based payment model with two components: a capitation for all GP care and a shared savings/losses model pertaining to total healthcare spending.^1^ Both components are explained in detail below.

#### Capitation for primary care spending (the ‘all-in tariff’)

The capitation payment – in the contract known as the ‘all-in tariff’ – replaces the entire three-segment model. In practice, this implies that the capitation fee in S1 is expanded to cover all services falling under the three segments as described in Sect. "Regular payment model for Dutch GPs: the three-segment model". The capitation is based on the care organization’s historical spending and adjusted annually for inflation.

This component of the AIC has three interrelated goals. First, to reduce the complexity and administrative burdens for GPs. For example, GPs no longer need to register and submit many detailed separate claims to the insurer. The second goal is facilitating patient-centered care. For example, GPs are no longer bound by consultations with a fixed time window when more time is needed. Also, they can more easily introduce new forms of care such as e-health that under the existing payment model does not fall under a billable code. A final goal is to stimulate cost-consciousness by removing the volume incentive, as GPs are no longer paid per individual consultation, diagnostic test, or medical treatment.

#### Shared savings/losses component

The second component is an updated version of the pilot program and encompasses a shared savings/losses model in which the participating primary care organization bears financial accountability for total medical spending and quality of care within their population of registered patients. This includes spending for the full set of curative services covered by basic and supplementary health insurance (except for adult dental care), including services from other providers such as hospitals. Under the AIC, the participating primary care organization undergoes an annual retrospective reconciliation process in which its population’s average spending is compared to a spending target. The care organization shares in the savings if spending falls below the target, and shares in losses if spending exceeds the target.

Spending on primary care services by the participating primary care organization was measured using the capitation payment (all-in tariff). This payment limited opportunities to realize shared savings within primary care itself, as GPs could not generate savings by reducing primary care use alone. Therefore, any savings/losses related to primary care are driven by the indexation of the capitation payment rather than by a behavioral response of the GPs. The shared savings incentives were largely designed to realize spending reductions in other spending categories, such as medical specialist care and pharmaceutical care via changes in referral practices or prescription behavior.

The size of the care organization’s payout/penalty depends on four factors: the difference between claims spending and the spending target, the performance on a set of quality indicators, the sharing rate, and a payout/penalty maximum. At the start in 2017, the AIC included 34 quality indicators, which can be divided into three categories: chronic disease management, patient satisfaction, and efficient prescribing of medication. These quality indicators were further developed during the program, and over the years, indicators have been removed and added. The spending target is calculated as a three-year weighted average of the care organization’s own historical spending, trended forward using the growth rate in healthcare spending of patients registered at non-participating GP practices. The target is corrected for patient characteristics that are predictive of spending (based on variables included in the Dutch risk equalization model for health insurer payment) [30]. For reasons of confidentiality, no details can be given on the sharing rate and payout/penalty maximum, but it should be understood as a relatively small percentage of total provider revenue. To account for statistical uncertainty resulting from the high variability in healthcare spending across individuals, a savings/losses threshold was established. Only when claims spending deviates by more than 2% of primary care spending (i.e., the all-in tariff), the financial result will be considered a saving or loss to be shared. To shield the primary care organization from excessive financial risk, a stop-loss arrangement caps its financial liability at €22,500 per patient per year.

The shared savings/losses component of the AIC has two goals. First, to prevent cost-shifting behavior by providing an incentive for making cost-conscious decisions in referring patients to specialist care, and by stimulating efficient substitution of hospital care for primary care. Second, to monitor and reward quality of care.

## Data and methods

In this section, we describe our data and methodology designed to estimate the AIC’s impact on spending, quality, and provider experiences 2.5 years after implementation. We combined different data sources and methods. In short, for the impact on spending we used a difference-in-differences approach using individual-level claims data to compare the spending of patients registered with participating GP practices against a control group of patients of non-participating GP practices, before and after the introduction of the AIC. The analysis of quality of care is based on a before-and-after evaluation of performance on quality indicators that are part of the shared savings/losses component, specifically focusing on chronic care management and patient satisfaction. Finally, we conducted focus groups with representatives from the health insurer and the participating primary care organization, including GPs. The aim of these focus groups was to gain insight into (i) how the quantitative results (if any) regarding spending and quality indicators could be explained or interpreted, and (ii) any other perceived impacts of the AIC.

### Impact on spending

#### Study population

To assess the impact on spending, we analyzed detailed patient-level claims data obtained from the involved health insurer. We used data over the period 2015–2019 from patients who were continuously insured at the insurer during this period. These patients were registered with either a GP participating in the AIC (intervention group) or a GP not participating in the AIC (control group). Specifically, the control group consisted of a 20% random sample of patients insured at the health insurer and who were registered with a GP that did not participate in the AIC. Patients who switched GP-practice during the study period were excluded. The total study population included 3,635 GPs and 212,251 patients. Within this population, the intervention group comprised 20 GPs with 16,425 registered patients. The GP-practices participating in the AIC are located in 7 of the 12 provinces in the Netherlands.^2^ We excluded GP practices that participated in the 2015 shared-savings pilot between the insurer and primary care organization, in which a shared-savings contract was added to the three-segment model [17]. These practices did not participate in the AIC intervention in 2017.

For each patient in our sample, the data contain information on all insurance claims under the contract, categorized into 13 spending categories including prescription medication, hospital care, GP care, and diagnostic services like laboratory tests. Additionally, the data include details about patients' socio-demographic characteristics and indicators of chronic illness (explained below).

#### Spending outcomes

We analyzed changes in total quarterly claims spending per patient, summed across the 13 subcategories. To mitigate the impact of outliers, before analysis we capped quarterly spending at the 95th percentile of the spending distribution [17]. In addition to total spending, we also separately examined the impact on three major spending subcategories: GP and multidisciplinary primary care, pharmaceutical care, and medical specialist care.

#### Patient characteristics

To account for differences in the characteristics of patients treated in the intervention and control group, we used patient-level administrative data obtained from the insurer. For all patients in our sample and for each quarter in the study period, we observe age (in 5-year bands), gender, socioeconomic status (based on age, income, education, and occupation), indicators for chronic illness based on prior use of prescription medication (i.e., pharmacy cost groups, PCGs) and diagnoses from prior hospital admissions (i.e., diagnostic cost groups, DCGs), and yes/no having supplementary insurance. As GPs can influence prescription medication and hospital admissions, we fixed the chronic illness indicators (i.e., PCGs and DCGs) on the value in 2017, which is the most recent pre-intervention year. We excluded patients from the analyses who had missing values for any of these variables (N = 60).

#### Difference-in-differences model specifications

To determine the effect of the AIC on the spending outcomes, we used a difference-in-differences (DD) design. In a DD-design, trends in an outcome before and after the intervention are compared between an intervention and control group. Using this design, we aimed to control for unobserved variables potentially leading to inherently different outcome trajectories and biased results [31]. The specification of our DD model is as follows:

1 $${Y}_{igq}={\beta }_{0}+{\delta }_{g}+{\alpha }_{q}+{\beta }_{1}{Post}_{q}*{Intervention}_{g}+\tau {X}_{i}^{T} {+\varepsilon }_{igq}$$

${Y}_{igq}$ refers to the spending of patient *i* enrolled at GP *g* in quarter *q*. ${\beta }_{0}$ is the constant and parameter ${\delta }_{g}$ denotes an intervention group fixed effect. Parameter ${\alpha }_{q}$ denotes quarter fixed effects which account for time trends common to both groups, and which also capture the post-intervention time effect. The variable ${Post}_{q}*{Intervention}_{g}$ is an interaction term between yes/no quarter *q* is in the post-intervention period and yes/no GP *g* is part of the intervention group. The associated parameter ${\beta }_{1}$ is the DD estimator and measures the effect of the AIC on the relevant spending outcome. ${X}_{i}^{T}$ is a row-vector of the abovementioned patient characteristics. Finally, ${\varepsilon }_{igq}$ refers to the error term. We used heteroscedasticity-robust standard errors clustered at the GP-level to account for correlation in outcomes between patients enrolled at the same GP, and for serial correlation within GP clusters. In our main analysis we used a Generalized Linear Model (GLM) with a normal distribution and a log link, as is common in the literature [32–34]. In addition, because we capped quarterly spending at the 95th percentile, expressing the effects in percentage terms has a more intuitive interpretation than the effects in euros.

#### Testing for parallel pre-intervention trends

A key assumption underlying the DD design is that the trends in the outcome variables for the intervention and control group as observed pre-intervention would have continued in the same way post-intervention if the intervention would not have been implemented. This is also referred to as the ‘parallel trends assumption’. We assessed the plausibility of this assumption in two ways: (i) visual inspection of the trends in outcome variables over time for both groups, and (ii) a formal falsification test. In this test, deviations in the common trends between intervention and control group in the pre-intervention period are statistically investigated by adding 'leads' and 'lags' to the regression model [31]. If the lead effects are jointly significant, this implies a deviation from the common trend, indicating a violation of the parallel trends assumption. For this falsification test we used the following model:

2 $${Y}_{igq}={\beta }_{0}+{\delta }_{g}+{\alpha }_{q}+\sum_{q=2015Q2}^{n}{\beta }_{q}{Time}_{q}*{Intervention}_{g}+\tau {X}_{i}^{T} {+\varepsilon }_{igq}$$

${Time}_{q}*{Intervention}_{g}$ refers to interaction terms of dummy variables indicating for each quarter *q* in the study period whether GP *g* is part of the intervention group or not. The parameters ${\beta }_{q}$ are the DD falsification test estimators and measure the (placebo) effect of the intervention for each quarter in the study period, where the reference quarter is 2015Q1 (i.e., the earliest available quarter). By means of a joint F-test on lead effects (i.e., pre-intervention quarters) we determined whether the pre-intervention trend of the intervention group deviated significantly from the pre-intervention trend of the control group, at a 5% significance level.

#### Additional analysis and robustness checks

To assess whether the AIC’s impact evolved over time, we conducted an additional analysis focusing on claims spending during the first 1.5 years of the intervention. This was done by excluding all claims spending incurred after the fourth quarter of 2018. In addition, we performed several robustness checks. First, we estimated the impact on total spending using an alternative specification of the control group, consisting of patients residing in the same region as patients registered with participating GPs but who are registered with a non-participating GP. With this ‘local’ control group we ensured comparable market conditions (e.g., in terms of hospital prices) between both groups, which are partly shaped by the regional healthcare purchasing teams of the involved insurer [17]. Secondly, to evaluate whether our estimates would change due to by spending on end-of-life care, we included the claims spending of patients who died during the study period. Third, to test the sensitivity of our results to model specification we re-estimated our model using a GLM with a normal distribution and an identity link. Fourth, to assess whether fixing the chronic illness indicators (i.e., PCGs and DCGs) at the 2017 values influenced our results, we re-ran our model but then with the chronic illness indicators fixed at 2016 [10]. Finally, we also calculated the AIC's effects on total uncapped spending.

### Changes in quality

#### Study period and quality indicators

Our analysis of changes in quality indicators covered the period 2016–2019 and focused on the indicators used in the AIC's shared savings/losses component (see Sect. 2.4.2). These data came from the involved primary care organization and comprised 11 indicators related to patient satisfaction and 13 indicators for chronic care management for DM2 and COPD. We excluded indicators lacking pre-intervention data and those with data available for only one post-intervention year. Patient satisfaction was measured via a survey distributed to all registered patients who visited a GP during a performance year. For the 11 indicators, we obtained the average score across all participating GPs as well as the standard deviation and the annual number of administered surveys from 2016 to 2019. For chronic care management, we obtained annual organizational-level scores for eight DM2 indicators and five COPD indicators for 2016–2018, with data for three indicators also available for 2019. Unlike patient satisfaction data, chronic care management data did not include measures of dispersion.

#### Analysis

Due to the lack of data for patients of non-participating GPs we were restricted to conducting a pre-post evaluation, which prohibits any causal interpretation of changes over time. For the patient satisfaction indicators, we employed a weighted t-test to determine whether there was a statistically significant difference in the average scores between the post-intervention period (2017–2019) and the pre-intervention period (2016). We used this test because of the data's aggregate nature and the unequal sample sizes and variances across years. To evaluate how the average differences evolved over time, we also compared the average scores of 2019 directly with those of 2016.

For the chronic care indicators, we compared the average scores from the pre-intervention period (2016) with those of the post-intervention period (2017–2018, and for three indicators also 2019). Again, we assessed whether these changes showed an increasing or decreasing trend over time. Due to lacking measures of dispersion around the average scores, we were unable to evaluate the statistical significance of differences in indicator scores before versus after the intervention.

### Focus groups

Between March and June 2023, we conducted two focus group sessions of two hours, each with representatives of the involved health insurer and primary care organization. The participants were selected based on their involvement in the development and/or implementation of the AIC and recruited via the AIC project managers of the two organizations. As these participants were directly engaged in the initiative, they may have had a more favorable view of the AIC and the results should therefore be seen as descriptive rather than causally attributed to the AIC. Participants in focus group 1 (on-site) included three GPs, a project manager, and an account manager from the primary care organization. In focus group 2 (online), a project manager and a senior intelligence analyst from the health insurer, and a project manager and an account manager from the primary care organization participated. The focus groups concentrated on two central questions: (i) How can the quantitative results (if any) regarding spending and quality indicators be explained or interpreted? (ii) What other impacts, if any, have you observed from the new payment model, including but not limited to patient centered care, innovation, and administration? Each focus group commenced with a presentation by author TG on the results of the quantitative evaluation. Subsequently, the group discussion was launched using a semi-structured topic list. Authors FE and DC took the lead during this discussion, posing specific and follow-up questions, while author TG observed and took notes.

The focus groups were conducted in accordance with NCCRI guidelines, with prior approval from the Ethical Review Committee (ETH2223-0329). Informed consent was obtained from all participants. Audio recordings were made and these recordings were transcribed verbatim. The data were coded and thematically analyzed independently by all three authors, incorporating both inductive and deductive approaches and using five predefined themes: (1) impact on claims spending, (2) changes in quality indicators, (3) workplace organization and care delivery, (4) digitalization and innovation, and (5) administrative burden. The main findings were discussed by the authors in a dedicated meeting, resulting in a single document containing key findings. This document was then shared with the focus group participants for a member check, leading to a few minor changes.

## Results

### Descriptive statistics

Table 1 shows some descriptive statistics for the intervention and control group for the pre-intervention period (i.e., Q1 2015 through Q2 2017).

**Table 1:** Descriptive statistics of patients in intervention and control group in the pre-intervention period (January 2015 through June 2017)

Characteristic	Intervention group	Control group	p-value*
Insured (N)	16,425	195,826	
Mean age (years)	43.7	44.8	0.000
Male sex (%)	49.9	50.0	0.920
Indicators for morbidity (%) ‡			
PCG	19.0	18.1	0.368
Asthma and COPD	4.8	4.5	0.764
Diabetes Mellitus type 1 and 2	5.5	4.6	0.368
DCG	11.3	11.9	0.549
Uptake of supplementary insurance (%)	84.0	86.9	0.003
Socioeconomic Status (%) ^#^			
High	23.8	26.9	0.002
Medium	49.6	53.4	0.000
Low	26.6	19.7	0.000
Individual Quarterly Claims Spending (€) §			
Total (uncapped)	615	559	0.000
Total	351	326	0.000
GP care	53	46	0.000
Pharmaceutical care	50	48	0.000
Medical specialist care	125	123	0.000

‡ Pharmacy cost groups (PCG) and diagnosis cost groups (DCG) are indicators of chronic illness that are used in the Dutch risk equalization model that is used for calculating annual risk-adjusted capitation payments for health insurers. PCGs are based on prior use of outpatient and inpatient prescription medication and DCGs are based on diagnoses of prior hospital admission [30]

^#^ Socioeconomic status is based on age, education, income and occupation

* P-values for the differences between the two groups were calculated using an independent t-test for continuous outcome variables and a two-proportion z-test for binary outcome variables

§ Spending categories are capped at the 95th percentile of the spending distribution (unless indicated otherwise)

On average, the intervention group was younger (43.7 vs 44.8 years, p < 0.01) and had a lower socioeconomic status (24.6% vs 19.7%, p < 0.01) than the control group. The percentage of males and the prevalence of chronic lung disease were similar in both groups. Total individual-level quarterly capped claims spending is €351 for the intervention group and €326 for the control group (difference = €25, p = 0.000), consisting of 15% primary care, 14% pharmaceutical care, 36% hospital care, and 35% other types of healthcare spending (e.g., paramedical or mental health care).

### Adjusted trend in spending

**Fig. 1:**
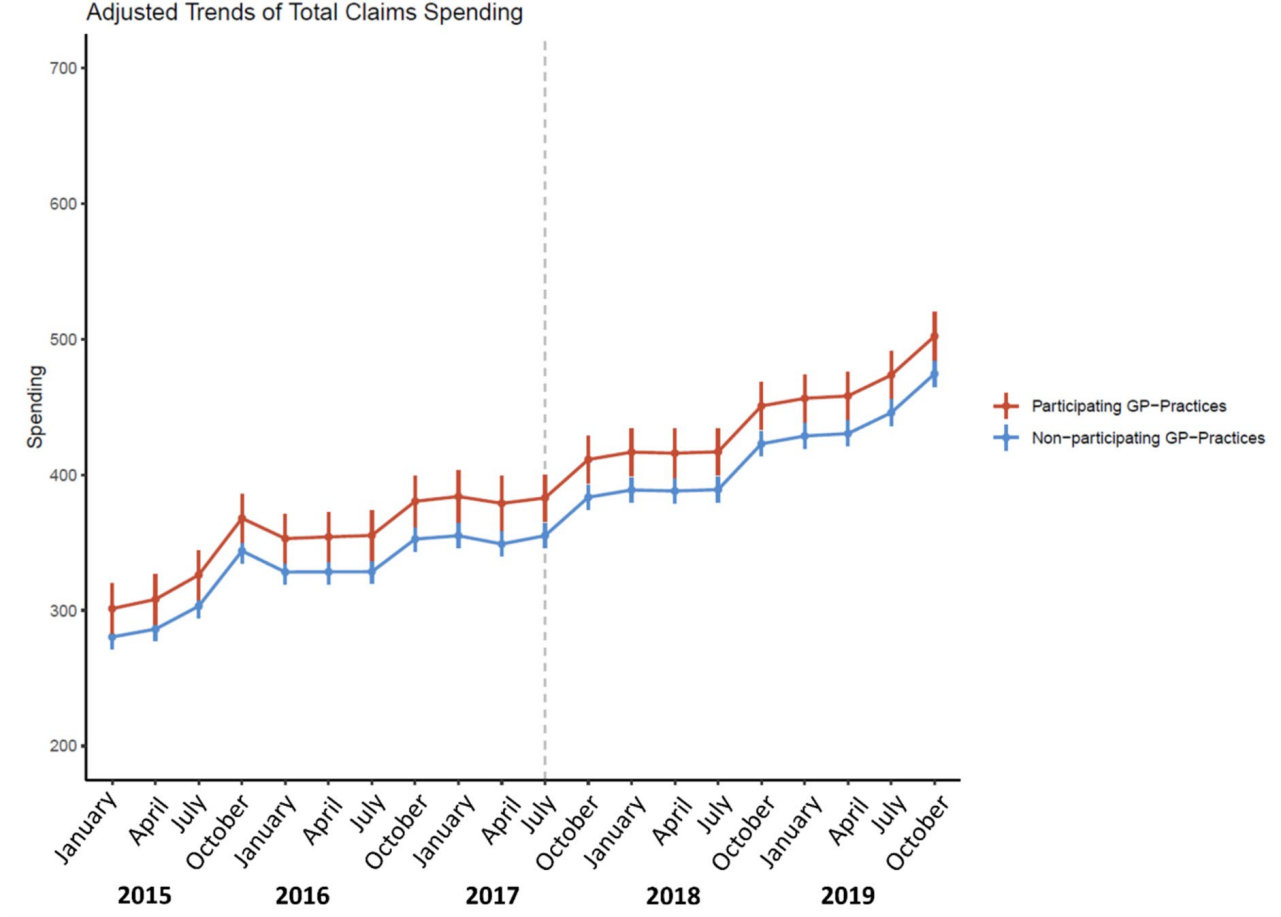
Adjusted trends of average total claims spending per patient in euros (capped at the 95th percentile). Note: The figure shows the trends in the average total claims spending per patient in euros over the entire study period for the intervention and control group. Vertical lines indicate 95% confidence intervals. The dashed line indicates the start of the AIC on July 1, 2017. Estimates are adjusted for intervention group and quarter fixed effects, as well as for patient characteristics

**Fig. 2:**
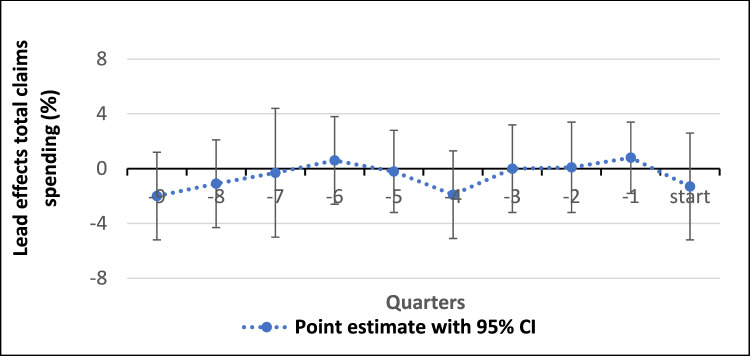
Individual lead effects to test for the assumption of parallel trends between study groups in the preintervention period for average total claims spending (%)

Figure 1 shows the adjusted trend in total claims spending per patient per quarter (capped at the 95th percentile). All estimates are adjusted for intervention group and quarter fixed effects, as well as patient characteristics as described in Sect. "Patient characteristics". Visual inspection suggests that the trends in total spending for the two groups were parallel in the pre-intervention period. This was confirmed by the falsification test, which showed that the lead effects were neither individually (see Fig. 2 in Appendix B) nor jointly statistically significantly different from zero (p = 0.642).

**Fig. 3:**
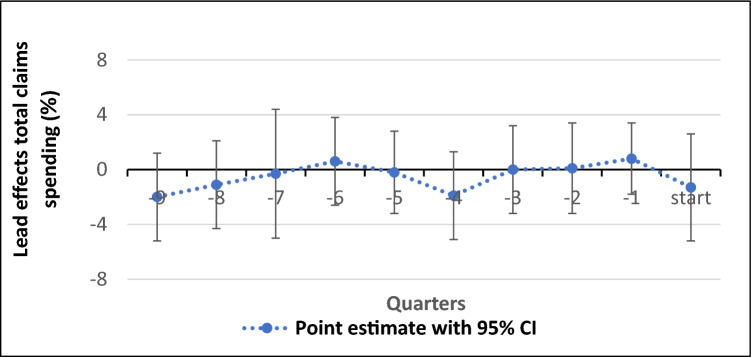
Individual lead effects to test for the assumption of parallel trends between study groups in the preintervention period for average claims spending on GP and multidisciplinary care (%)

**Fig. 4:**
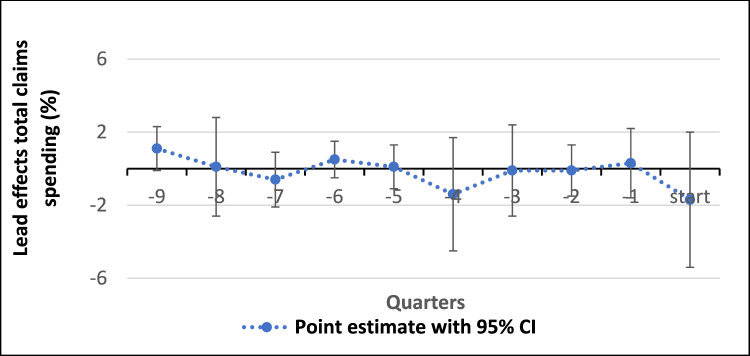
Individual lead effects to test for the assumption of parallel trends between study groups in the preintervention period for average claims spending on pharmaceutical care (%)

**Fig. 5:**
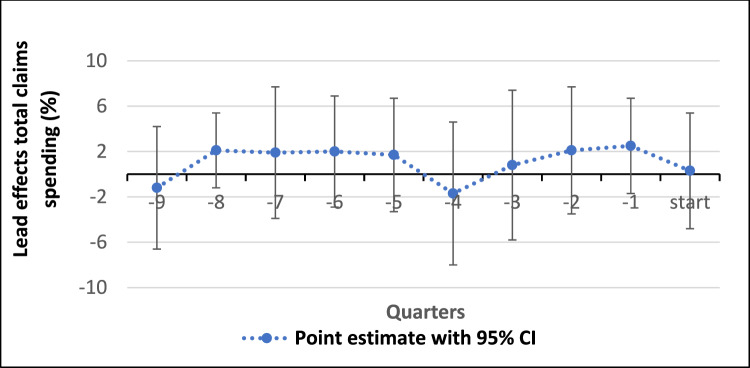
Individual lead effects to test for the assumption of parallel trends between study groups in the preintervention period for average claims spending on medical specialist care (%)

Figures. 3, 4, 5 in Appendix B show the results of the falsification tests of three subcategories of healthcare spending: GP and multidisciplinary primary care, pharmaceutical care, and medical specialist care. The falsification tests indicate that the lead effects of pharmaceutical and medical specialist care spending were neither individually nor jointly statistically significantly different from zero (p = 0.559 and p = 0.287, respectively). In contrast, as shown in Fig. 3, the lead effects of spending on GP and multidisciplinary primary care were jointly significant (p = 0.000). On average, GPs in the intervention group experienced significantly lower spending growth in this category in 2016Q2 and 2016Q3, followed by significantly higher growth in 2017Q1. Thus, the results for this spending category should not be interpreted as a causal effect of the AIC.

### Impact on spending

Table 2 reports the DD estimates of the AIC’s effects on claims spending 2.5 years after implementation. On average, total claims spending per patient per quarter grew 1.2% less for GPs participating in the AIC compared with GPs in the control group. However, this estimate is not statistically significant (p = 0.476), was found to be robust to alternative model specifications, and did not substantively change over time (see Table 3 in Appendix C).

**Table 2:** Difference-in-differences estimates for spending outcomes for the first 2.5 years after implementation of the AIC

Outcomes §	Difference-in-Differences (95% CI)	p-value
Total claims spending (%)	− 1.2 (− 4.7 to 2.2)	0.476
GP and multidisciplinary care spending (%)	− 10.2 (− 11.9 to − 8.4)	0.000
Pharmaceutical care spending (%)	− 1.3 (− 3.2 to 0.7)	0.198
Medical specialist care spending (%)	− 0.0 (− 3.3 to 3.2)	0.995

*CI* Confidence Interval, *AIC* all-in contract

§ Estimates are adjusted for intervention group and quarter fixed effects, as well as for patient characteristics. Standard errors clustered at the GP practice-level

We found that uncapped total spending increased by 3.3% (p = 0.156) compared to the control group (see Table 3 in Appendix C). However, the parallel trends assumption was not met (p = 0.000), suggesting that this result may reflect outliers disproportionately affecting the smaller intervention group and/or pre-existing deviations in spending trends before the intervention.

We found a substantial and significant decrease in spending on GP and multidisciplinary care post-intervention (− 10.2%, p = 0.000). However, although spending trajectories in this category did not decrease prior to the intervention, the parallel trends assumption did not hold. Thus, we cannot attribute this decrease in spending to the AIC. For spending on pharmaceutical care and medical specialist care, we observe small, statistically insignificant decreases in spending of − 1.3% (p = 0.198) and – 0.0% (p = 0.995), respectively.

### Changes in quality indicators

Table 4 in Appendix D details the changes in average scores on the patient satisfaction indicators, comparing the 1.5 years before implementation of the AIC (2016 and 2017Q1-Q2) with the 2.5 years thereafter (2017Q3-2019). Overall, the results indicate that patient satisfaction with GP care did not change much during the study period. Nonetheless, small significant changes were observed in three individual indicators: ‘the ease with which you could make an appointment’ (− 1.2%, p = 0.005), 'the time within which you could make an appointment' (− 1.7%, p < 0.000), and 'the extent to which the information during the consultation was clearly stated' (+ 0.8%, p = 0.017).

In an additional analysis comparing the average scores from 2016 with those of 2019 (Appendix D Table 5), we found a more pronounced decrease in the two indicators about making appointments (− 1.9%, p < 0.001, and − 2.7%, p < 0.001, respectively) and a significant decrease in a third indicator, 'telephone accessibility' (− 1.3%, p = 0.005). This analysis also revealed significant increases in two additional indicators: 'expertise of employees' and 'extent to which information is understandable' (+ 1.0%, p = 0.010, and + 1.1%, p = 0.002, respectively).

Table 6 in Appendix D reports changes in chronic care management indicators. For DM2, we found no overall change between 2016 and 2017–2019 (average change across all eight indicators = − 0.1%), with individual indicators showing both decreases and increases, ranging from − 3.3% to + 2.0%. Regarding COPD, our findings show a modest overall increase in quality indicator scores, with an average improvement of + 2.4% across all five indicators and increases for individual indicators ranging from + 1.4% to + 3.5% (testing these changes on statistical significance was not possible).

### Focus groups

The focus groups provided additional information on the following five themes: (1) impact on claims spending, (2) changes in quality indicators, (3) workplace organization and care delivery, (4) digitalization and innovation, (5) administrative burden.

Participants expressed dissatisfaction with the lack of reductions in claims spending, though they were not surprised by this outcome. They suggested multiple explanations for this lack of effect. First, participants noted that although both the study and the calculations of shared savings assess spending at an aggregated level, most projects aimed at achieving shared savings concentrate on specific medical services or treatments, such as ultrasound procedures. A participant suggested that savings may have been realized in more narrowly defined spending categories. Second, the financial incentives in medical specialist care remained unchanged under the AIC. In the Netherlands, virtually all hospitals operate under a budgeted contract (either a global budget or a cost-per-case contract with expenditure cap) [35], potentially nullifying the impact of endeavors to reduce spending through efficient referral practices by GPs. Third, participants noted heterogeneity among participating GP-practices, particularly in the medical conditions targeted by projects aimed at realizing savings and in degree of staffing shortages. Some shared savings projects may have been more successful in achieving spending reduction than others, and a shortage of staff in certain practices may have led to prioritizing the maintenance of operational capacity over optimizing referral efficiency, for instance. Finally, while participants mentioned efforts to reduce pharmaceutical spending by enhancing prescribing efficiency, they also noted obstacles such as frequent staff turnover and ingrained prescribing habits that may take a long(er) time to change.

Regarding the second theme (i.e., changes in quality indicators), participants noted that significant efforts to improve chronic care management for DM2 had already been implemented well before the AIC. They suggested that COPD care offered greater potential for improvement, as bundled payments for COPD care under the three-segment model had been introduced only one year prior to the AIC’s implementation. This could explain the greater emphasis on COPD indicators in the focus group discussions. Participants linked improvements in patient satisfaction indicators, such as ‘staff expertise’ and ‘clarity of information’, to AIC-facilitated hospitality training and campaigns across the organization. In addition, they noted that the decrease in patient satisfaction regarding accessibility may stem from (nation-wide) staffing shortages and could reflect perceived rather than actual accessibility changes.

With respect to workplace organization and care delivery, focus group participants mentioned that the population-based payment component (the all-in tariff) provides GPs with greater freedom to organize and deliver care at their own discretion, which positively contributed to their experienced job satisfaction. For example, regarding task reallocation, GPs were able to support practice assistants in answering questions from patients. These activities would not have fallen under a billable code in the regular payment model, while under the AIC there are no adverse financial consequences. According to the respondents, this resulted in fewer and more efficient GP consultations.

Fourth, participants noted that the AIC – specifically the removal of billable codes for GP care – enhanced the adoption and use of digitalization and innovative ways of working. For example, the primary care organization was able to invest in secure video consultations with patients, e-consultation of medical specialists, training on effective medication prescribing, lean working, reflection on referral behavior, and a digital portal for appointment scheduling. Under the regular payment model, these activities could have resulted in less revenue.

Finally, participants – including the GPs – reported that the AIC has resulted in a modest reduction of GPs’ administrative burdens. For instance, during longer consultations, individual billable items like the use of nitrogen no longer require separate registration, and there has been a decrease in internal monitoring of registration accuracy. Other representatives from the primary care organization experienced a significant reduction in their administrative workload because of the AIC, particularly in managing individual invoices for the insurer.

## Discussion

### Summary and discussion of main findings

This study evaluated a primary care population-based payment initiative (the All-In Contract: AIC), focusing on its impact on healthcare spending, changes in patient satisfaction and chronic care management indicators, and provider experiences. We find no evidence for a reduction in total spending attributable to the AIC. Changes in patient satisfaction and chronic care management were small and mixed. Representatives of the primary care organization conveyed increased perceived flexibility and autonomy, greater adoption of digital solutions and care delivery innovation, alongside a reduction in administrative burdens. Below we discuss these findings, drawing also from the insights derived from the focus groups.

There are several potential explanations for the absence of an effect on total claims spending. First, there may have been heterogeneity among participating GP practices which could have influenced the AIC's impact on total spending at the broader organizational level. As highlighted by the focus group participants, there were marked differences between practices regarding factors like staffing shortages and in the medical conditions targeted in response to the shared savings component of the AIC. These differences may have shifted the focus away from generating savings and consequently diluted the impact on spending. Given the lack of GP-practice characteristics and the limited size of individual participating GP practices, it was not feasible to estimate treatment effects at the practice level in our impact analyses.

Second, the AIC was guided by quadruple aim, meaning that it aimed to achieve more than reduced spending growth. At least in the short run, the additional goals of the AIC related to the replacement of the three-segment model with an all-in tariff per enrolled patient may have diverted GPs’ attention away from efficient substitution and referrals to other objectives like patient-centered care and innovative care delivery practices like e-health. This might be one reason why our results contrast with the results found in the pilot version of the AIC, which found significant reductions in total claims spending when only a shared savings model was added to the prevailing three-segment model [17]. Relatedly, our study period comprising 2.5 years post intervention may have been too short for significant effects on spending to materialize.

Third, the AIC may have had limited potential to reduce total spending. The regular three-segment payment model already incorporates aspects of population-based payment (i.e., the fixed quarterly enrollment fee) and bundled payments for selected chronic conditions, which may have mitigated the potential for spending reductions. Additionally, the combination of several design features of the AIC, such as the 2% band around the spending target, relatively low shared savings rates, and reductions in primary care utilization not contributing to realizing shared savings (because of the all-in tariff), likely dampened the incentive to achieve savings. Furthermore, focus group participants noted that the insignificant effect on medical specialist care spending could stem from GPs' limited influence on spending outside of primary care, despite their role as gatekeepers. Specifically, the fact that virtually all hospitals in the Netherlands operate under budgeted contracts with strong incentives for volume below expenditure caps, may hinder the ability of Dutch GPs to reduce medical specialist care spending through more efficient referral practices. This may explain why, in contrast to the payment initiative evaluated here, population-based payment models in the US have shown substantial significant spending reductions [10, 11, 13, 15]. Two key contextual differences may have contributed to this difference in impact: i) fee-for-service being the prevailing payment model in the US (and thus in control groups used in evaluation studies), potentially offering greater opportunities for improvement, and ii) integrated provider entities such as Accountable Care Organizations (ACOs) forming intervention groups in evaluation studies (instead of GP practices in our study), which likely have more influence on spending across a broader spectrum of care. Although we did find a significant relative decrease in spending on GP and multidisciplinary care (which GPs can directly influence), trends in the pre-intervention period were not parallel, preventing us from attributing this result to the AIC.

We observed a significant 10.2% decrease in primary care spending growth. This result aligns with the results of previous studies that linked the introduction of capitation to reduced spending [20–22]. While the parallel trends assumption for this spending category was rejected, the effect is unlikely to be entirely due to statistical noise. As primary care spending is measured by the capitation payment (i.e., the all-in tariff), it is likely that the indexation rate applied to this payment was lower than that for control practices. In addition, spending patterns in the control group could have diverged from those in the intervention group in three ways: increased utilization of (costly) primary care services, higher prices for these services, and/or the introduction of additional billable services for primary (GP) care. Unfortunately, the introduction of the all-in tariff eliminated the collection of claims data on billable primary care activities for the intervention group, rendering such data unavailable for evaluation. Consequently, while we could assess patterns in primary care spending, it was impossible to distinguish between changes driven by utilization, pricing, or both. It is worth noting that while an effect of −10% seems large, in absolute terms it is quite modest given that primary care spending accounts for only 15% of total capped claims spending in our data.

We observed mixed and modest changes in indicators of patient satisfaction and chronic care management, similar to the pilot study [17]. However, due to the lack of data from non-participating GPs, it is unclear whether it is unclear whether and to what extent these changes can be attributed to the AIC. Notably, focus group participants suggested that larger improvements in COPD care compared to DM2 reflect the greater improvement potential for COPD, as DM2 care had already been a focus of prior initiatives. Focus group participants attributed significant improvements in specific patient satisfaction indicators, such as communication and staff hospitality, to training sessions and campaigns supported by the capitation component of the AIC. However, perceived access indicators, such as appointment availability, significantly declined. This finding is consistent with prior work that has associated the introduction of capitation with reduced access to primary care services [20–22]. In addition, focus group participants suggested that the decline in perceived accessibility may also have been a result from nationwide staffing shortages. Comparing these findings internationally is challenging due to differences in the quality indicators used in other population-based payment evaluations [10, 15].

Although the shared savings component of the AIC showed no measurable impact on spending growth or quality indicators, the capitation component was designed to improve provider flexibility, autonomy, and reduce administrative burden. Focus group participants confirmed improvements in these areas. Given the nationwide shortage of GPs in the Netherlands [36], models like the AIC may still represent progress over the three-segment model by improving job satisfaction and addressing staffing challenges. However, the selection criterion of focus group participants could have resulted in a more favorable view of the intervention’s outcomes, and the results should therefore be seen as descriptive rather than causally attributed to the AIC.

### Limitations

Our findings must be interpreted in the light of several limitations. A first potential limitation is the selection bias arising from the AIC being targeted at a specific nationally operating primary care organization. Specific characteristics of this organization, such as capacities related to data collection and analysis, may not be generalizable to other (smaller) provider entities. Although this concern is somewhat mitigated by parallel pre-intervention trends in total spending, the estimates with respect to spending should be interpreted as the Average Treatment Effect on the Treated (ATT).

Second, as the parallel trends assumption did not hold for the outcome variable ‘spending on GP and multidisciplinary primary care’, the results for this outcome cannot be causally attributed to the AIC and should be interpreted with caution.

Third, there were several limitations related to data availability. For example, quality indicator scores were not available for a control group, precluding causal inference. Additionally, the introduction of the capitation meant the insurer stopped collecting data on billable primary care activities, rendering these data unavailable for our evaluation. Furthermore, we did not have access to data that would allow us to calculate net program savings, such as information on the implementation and maintenance costs of the intervention, and data on (re)payments of shared savings or losses. Also, due to the COVID-19 pandemic we limited our estimates of changes in spending and quality to the first 2.5 years post-implementation (up to 2019). Finally, as with any study involving focus groups, selective participation could have introduced bias in the sense that the opinions and experiences shared during the focus group sessions may not fully capture the broader and diverse viewpoints of all stakeholders. Additionally, recall bias may have influenced our findings given that participants were asked to reflect on experiences from several years prior.

### Implications

Our study offers two valuable lessons for policymakers, payers, and healthcare providers considering payment reform. First, given the multifaceted goal of population-based payment, a broad approach to evaluation that goes beyond the standard quantitative effects on spending and quality indicators is crucial for effective decision-making on payment reform. Although in its first 2.5 years the AIC did not reduce spending and changes in quality indicators were mixed and limited, focus group findings suggest that the initiative has reached more success on other goals, particularly provider experiences due to reduced administrative burdens, more flexibility, and more room for innovative ways of working. One perspective could be that these positive experiences have been achieved without reductions in quality (insofar measured) and increases in spending. Nevertheless, despite our broad approach, this evaluation provides an incomplete picture, particularly due to a lack of data on quality performance (e.g., no control group) and care utilization (e.g., no information on billable primary care services). To be able to obtain a complete picture on the impact of payment reform in relation to its goals, stakeholders embarking on payment reform should devise a comprehensive evaluation plan including data collection strategies and a long time horizon.

Second, before implementing a population-based payment model it is important to assess both the improvement potential for each of the stated goals and the extent to which this improvement potential can be realized through behavioral change of the participating care providers. The shared savings/losses component of the AIC aimed to reduce total spending while at least maintaining quality of care, a goal that was not achieved (at least not in the first 2.5 years). Relative to population-based payment initiatives in other countries which seem to have had more success in this regard, the limited impact of the Dutch AIC so far may have been a result from contextual factors such as a shortage of GPs and supporting personal, non-FFS control group, volume incentives in hospitals, and lacking integration between primary and secondary care. Achieving meaningful reductions in spending is likely to require financial accountability for larger, integrated provider organizations with influence over the entire continuum of care.

### Conclusion

In its first 2.5 years, the AIC had no significant effect on total healthcare spending growth. While GP care spending growth decreased by 10.2%, this is likely attributable to the indexation design of the capitation component rather than a change in the behavior of participating GPs. Changes in patient satisfaction and chronic care management indicators were mixed and modest, but due to the lack of data from non-participating GPs, their attribution to the AIC remains uncertain. Focus group participants highlighted improvements in provider flexibility, autonomy, and reduced administrative burdens, though these insights may be subject to seleQ5ction bias. To comprehensively evaluate population-based payment reforms, stakeholders should prioritize robust data collection, including metrics on population health, patient satisfaction, and provider well-being.

## Data Availability

The data supporting the findings of this study are available from Coöperatie Menzis U.A., the program’s health insurer. However, due to licensing restrictions, these data are not publicly available. Data may be accessed upon reasonable request and with permission from Coöperatie Menzis U.A.

## Appendix

Supplementary Appendix for “Changes in Spending, Quality Indicators, and Provider Experiences Following the Introduction of a Population-Based Payment Model in Dutch Primary Care: A Mixed Methods Evaluation”.

See Tables 3, 4, 5, 6

See Figs. 2, 3, 4, 5

## A. Shared Savings Pilot

In 2015, the insurer and primary care organization involved in the AIC initiated a pilot which added a shared-savings contract to the three-segment model. In the pilot, GP practices in a relatively small region in the Netherlands could share in savings on total healthcare spending relative to a preset spending target and conditional on achieving quality targets [17]. An evaluation of the pilot showed a significant reduction in total spending (-2%) after the first year, without changes in quality [17]. However, the pilot and its evaluation had several limitations. First, the shared-savings model was added to the three-segment model and did not replace it. Consequently, the drawbacks of the three-segment model largely remained, and the payment system as a whole became even more complex. Second, the pilot was implemented in a specific region and the results may not be representative for the rest of the country. Last, the evaluation only covered the first year of the pilot, while previous evaluations of population-based payment initiatives have shown stronger and more significant results when participants are exposed to new incentives over longer periods [10, 11, 15].

## B. Falsification test parallel trends assumption: individual lead effects

See Figs. 2, 3, 4 and 5.

## C. Results of additional analyses and robustness checks

See Table 3.

**Tabel 3:** Difference-in-Differences estimates of additional analyses and robustness checks for total health care spending†

Model	Difference-in-differences total claims spending	p-value
Base model (1)	− 1.2 (− 4.6 tot 2.2)	0.476
Regional control group (2)	− 1.4 (− 4.9 tot 2.2)	0.437
Include deceased (3)	− 1.2 (− 4.5 tot 2.2)	0.475
Identity link (€) (4)	− 1.1 (− 14.9 tot 12.7)	0.877
PCG/DCG base year 2016 (5)	− 1.4 (− 4.9 tot 2.2)	0.432
No cutoff 95th percentile (6)	3.3 (− 1.3 tot 8.2)	0.156
First 1.5 years (excluding 2019) (7)	− 1.7 (− 4.3 tot 1.1)	0.228

*CI* Confidence Interval, *AIC* all-in contract

† Estimates are adjusted for intervention group and quarter fixed effects, as well as for patient characteristics

## D. Results of changes in quality-of-care indicators

See Tables 4, 5 and 6.

**Table 4:** Changes in average scores on the patient satisfaction indicators, comparing the 1.5 years before implementation of the AIC (2016 and 2017Q1-Q2) with the 2.5 years thereafter (2017Q3–2019)

Quality Indicator	Mean Before %	Mean after %	Difference	P-value
Total	82.9	82.9	0.0	
Ease of making an appointment	83.1	81.9	− 1.2	0.005
Availability of appointment time	81.5	79.7	− 1.7	0.000
Telephone accessibility	80.0	79.3	− 0.6	0.138
Friendliness of staff	83.7	83.8	0.1	0.656
Attention given to the patient during the consultation	84.4	84.9	0.5	0.185
Extent to which healthcare providers try to understand the problem	85.2	85.7	0.5	0.188
Extent to which the patient can participate in decision making during the consultation	84.5	84.9	0.4	0.358
Level of expertise of staff	83.5	84.2	0.7	0.053
Extent to which the patient is informed	83.4	83.9	0.5	0.185
Clarity of information	84.8	85.6	0.8	0.017
The (provisional) effect of the consultation	78.1	78.0	− 0.1	0.767

** p* < *0,1, ** p* < *0,05, *** p* < *0.01*

**Table 5:** Changes in average scores on the patient satisfaction indicators, comparing the 1.5 years before implementation of the AIC (2016 and 2017Q1-Q2) with 2019

Quality Indicator	Mean before %	Mean 2019 %	Difference %	P-value
Total	82.9	82.8	− 0.1	
Ease of making an appointment	83.1	81.2	− 1.9	0.000
Availability of appointment time	81.5	78.8	− 2.7	0.000
Telephone accessibility	80.0	78.7	− 1.3	0.005
Friendliness of staff	83.7	83.9	0.2	0.596
Attention given to the patient during the consultation	84.4	85.0	0.6	0.121
Extent to which healthcare providers try to understand the problem	85.2	85.9	0.8	0.060
Extent to which the patient can participate in decision making during the consultation	84.5	85.1	0.6	0.158
Level of expertise of staff	83.5	84.5	1.0	0.010
Extent to which the patient is informed	83.4	84.1	0.7	0.077
Clarity of information	84.8	85.9	1.1	0.002
The (provisional) effect of the consultation	78.1	78.2	0.1	0.801

** p* < *0,1, ** p* < *0,05, *** p* < *0.01*

**Table 6:** Changes in average scores on chronic care delivery indicators, comparing the 1.5 years before implementation of the AIC (2016 and 2017Q1-Q2) with the 2.5 years thereafter (2017Q3-2019)

Domain	Quality Indicator	Mean before %	Mean after %	Difference %
Chronic Care	DM 2—LDL < = 2,5 (5Jr)—Dg < 80 ‡	59.0	61.0	2.0
	DM 2—Statins ‡	68.3	65.0	– 3.3
	DM 2—Kidney function	97.0	97.1	0.1
	DM 2—Urine	90.1	91.5	1.4
	DM 2—Smoking status	99.1	98.5	– 0.6
	DM 2—Smoking status active	18.9	17.8	1.1
	DM 2—Fundus	92.7	92.3	– 0.4
	DM 2—Foot exam	93.6	92.1	– 1.5
	DM 2—Total			– 0.1
	COPD—Inhalation technique	87.5	89.8	2.3
	COPD—Functioning ‡	93.0	96.5	3.5
	COPD—Exercise	93.3	96.0	2.7
	COPD—Smoking status	96.2	97.6	1.4
	COPD—Smoking status active	51.4	49.4	2.0
	COPD—Total			2.4

^‡^Indicator available in 2019.

## Footnotes

1 In 2015, the insurer and primary care organization launched a pilot in a small region in the Netherlands adding a shared-savings arrangement to the three-segment model [17]. GP-practices participating in this pilot did not participate in the AIC in 2017. See Appendix A for further details.

2 South Holland (11), Utrecht (3), Gelderland (2), North Holland (1), Groningen (1), Friesland (1) and Overijssel (1).
